# Spiritual Well-Being, Religiosity, Psychological Distress and Quality of Life in Adults with Cancer: A Cross-Sectional Study in a Romanian Oncology and Palliative Care Centre

**DOI:** 10.3390/cancers18172840

**Published:** 2026-09-02

**Authors:** Dana Sonia Nagy, Alexandru Catalin Motofelea, Sorin Saftescu, Vlad-Norin Vornicu, Razvan Gheorghe Diaconescu, Dan Ionel Orbulescu, Catalin Alexandru Pirvu, Serban Mircea Negru

**Affiliations:** 1 Doctoral School, “Victor Babes’’ University of Medicine and Pharmacy, Eftimie Murgu Square No. 2, 300041 Timisoara, Romania; dana.nagy@umft.ro; 2 Department of Palliative Care, OncoHelp Hospital Timisoara, Ciprian Porumbescu Street, No. 59, 300239 Timisoara, Romania; razvan.diaconescu@umft.ro (R.G.D.); serban.negru@umft.ro (S.M.N.); 3 Department of Oncology, Victor Babes University of Medicine and Pharmacy, Bd. Victor Babes No. 16, 300226 Timisoara, Romania; sorin.saftescu@umft.ro (S.S.);; 4 Center for Molecular Research in Nephrology and Vascular Disease, Faculty of Medicine, “Victor Babes” University of Medicine and Pharmacy, 300041 Timisoara, Romania; 5 Plastic Surgery Department, “Victor Babes” University of Medicine and Pharmacy, 300041 Timisoara, Romania; dan.orbulescu@umft.ro; 6 Department X Surgical Emergencies Clinic, “Victor Babes” University of Medicine and Pharmacy, 300041 Timisoara, Romania; pirvu.catalin@umft.ro

**Keywords:** spiritual well-being, psychological distress, religiosity, oncology, quality of life, cross-sectional study, FACIT-Sp-12

## Abstract

**Background:** Spiritual wellbeing is increasingly recognised as a key area of complex oncology and palliative care, but evidence from Eastern Europe is still limited. Understanding how spiritual well-being and religiosity are related to psychological distress and quality of life can inform holistic support interventions in cancer patients. **Objective:** To describe spiritual well-being, religiosity, anxiety, depression, and quality of life in a Romanian cancer cohort, and to examine associations between spiritual well-being and psychological distress. **Methods:** A cross-sectional study was carried out in 124 cancer-stricken adults at a regional cancer centre in Romania. Participants completed FACIT-Sp-12 (spiritual well-being), RCI-10 (religious belief), HADS (depression), and EQT QLQ-C30 (QoL). Multivariable linear regression was used to determine independent predictors of outcomes in patients. **Results:** Participants had a median age of 65 years (53–71) and 70 (56%) patients had metastatic disease. Religious involvement was high and spirituality related to disease was commonly reinforced, with 66 (53%). There were twice as many cases of clinical depression symptoms (28%) as anxiety symptoms (14%). A median QoL score of 51 (IQR: 37–64 points) was revealed by descriptive analysis of the EORTC QLQ-C30. In adjusted multivariable models, spiritual well-being was significantly and independently associated with lower anxiety (β = −0.25, *p* = 0.030) and depression (β = −0.29, *p* = 0.013), with models accounting for 11.5% and 10.8% of variance, respectively. Global quality of life was best explained by the model (R^2^ = 26.4%), with independent contributions from impaired ECOG performance status (β = −0.27, *p* = 0.006) and depressive symptoms (β = −0.22, *p* = 0.014), whereas religious commitment showed no independent association with any primary outcome. **Conclusions:** In this Romanian cohort, spiritual well-being, not religious adherence, remained independently associated with lower anxiety and depression, suggesting that spiritual well-being is associated with better psychological health among cancer patients. Religious affiliation has not been shown to be independent of any outcome, a difference with direct implications for the screening of spiritual care. Global quality of life, on the other hand, was independently associated with both physical functional status (ECOG) and depressive symptom burden, rather than with spiritual or religious factors, underlining that both functional decline and psychological distress remain central to cancer-related quality of life.

## Simple Summary

Cancer patients often face not only physical symptoms but also profound emotional and spiritual challenges. Understanding how spiritual beliefs and practices relate to mental health can help healthcare teams provide better support. This study examined 124 cancer patients in Romania to explore associations between spiritual well-being, religious commitment, anxiety, and depression. We found that patients with higher spiritual well-being experienced lower levels of anxiety and depression, with particularly strong links to reduced depression. Most patients reported that their illness had actually strengthened their spiritual beliefs and practices. These findings suggest that routinely assessing spiritual well-being in cancer care could help identify patients at risk for psychological distress and guide more complete, person-centered interventions that address both emotional and spiritual needs alongside medical treatment.

## 1. Introduction

The pathogenesis of cancer-related distress is complex, multidimensional, and driven by a constellation of neuro-immune interactions, behavioral risk factors, and psychosocial challenges [1]. Beyond the physical burden of illness, patients often face deep existential vulnerabilities. Current cancer care models emphasise that symptom management cannot be limited to the biological domain, but rather that existential distress is a distinct dimension of suffering that requires special attention to optimise the outcome of patients [2,3]. As a result, international guidelines increasingly recommend the inclusion of spiritual guidance as a standard of comprehensive symptom management in routine oncology practice [4].

Spiritual well-being (SpWB) has emerged as a critical construct in this context, distinct from physical health and emotional functioning. It is characterized not only by religious practice but by the presence of functional attributes such as meaning, peace, and a sense of connection [5]. Empirical evidence indicates that SpWB serves as a vital coping resource across diverse populations. For instance, Almeida et al. (2022) identified SpWB as a key driver of post-traumatic growth [6], while Quinto et al. (2022) found it central to the acceptance of illness [7]. In the palliative care setting, Biji et al. (2025) demonstrated that higher SpWB is strongly correlated with improved global quality of life (QoL) [8]. Conversely, Balboni et al. (2010, 2011) have shown that unmet spiritual needs are associated with diminished QoL, increased healthcare costs, and lower utilization of hospice services at the end of life [9,10]. Furthermore, a recent systematic review by Martinez-Calderon et al. (2025) indicates that spiritual well-being may even buffer the physical experience of illness, reporting a significant negative correlation between SpWB and cancer-related fatigue [11].

However, the literature suggests a critical nuance: religious commitment (adherence to rituals and dogmas) and spiritual well-being (the internalized sense of peace and meaning) are not interchangeable and may have divergent associations with mental health. While religiousness and spirituality often overlap, they are not identical. Grossoehme et al. (2020) utilized structural equation modeling to demonstrate that religious behaviors were not directly associated with reduced anxiety and depression unless they were mediated by a sense of meaning and peace [12]. Similarly, while Martinez-Calderon et al. found consistent associations between overall SpWB and reduced fatigue, the specific dimension of faith did not show a statistically significant correlation with symptom relief [11]. This aligns with findings by Schultz et al. (2017) [13], who noted that general distress screening tools often fail to identify spiritual distress, and that the “Peace” subscale of spiritual assessment tools is a stronger predictor of patient outcomes than the “Faith” subscale [13].

This distinction carries direct clinical implications: interventions targeting religious ritual alone may fail to address the existential peace patients actually need, yet whether this holds across cultural contexts remains poorly understood. Torres-Blasco et al. (2024) emphasized this in Latino populations, finding that SpWB served as a specific buffer against hopelessness and depression, distinct from general religiosity [14]. Similarly, Sastra et al. (2021) highlighted that spiritual needs and influencing factors vary significantly based on cultural and religious background, as seen in Indonesian Muslim populations [15].

Despite this growing recognition that cultural and religious background shapes the SpWB–distress relationship, systematic empirical data remain concentrated in North American and Western European settings, and notably in Latino and Asian populations; Eastern Europe, and Romania in particular, remains almost entirely unstudied [16]. Romania represents a unique cultural context characterized by a predominant Orthodox Christian tradition where religious observance is notably high. More than 86% of the population identifies as Orthodox, with high rates of church attendance and prayer serving as primary coping mechanisms for life-threatening illnesses [17]. Furthermore, Bacoanu et al. (2024) found that for Romanian palliative care patients, faith is perceived as a critical support system alongside family, yet the specific interplay between this high religiosity and psychological symptoms like anxiety remains complex [16]. It remains unclear whether the divergence between religiosity and spiritual well-being observed in secularized Western populations holds true in this specific setting, or if the high baseline religiosity offers a unique protective buffer against the psychological impact of advanced cancer.

This study addresses this critical gap by unifying the independent contributions of spiritual well-being and religious commitment to psychological distress and quality of life in a cohort of Romanian cancer patients, a population characterised by a uniquely high baseline religiosity, but which is not yet widely reported in the literature. By using different, validated measures for each construct in a single multivariable framework, we can directly test whether internalized spiritual well-being, rather than religious adherence, is more strongly associated with mental health and quality of life outcomes in this particular cultural context.

## 2. Materials and Methods

### 2.1. Study Design and Setting

This cross-sectional observational study was conducted at OncoHelp Hospital, a regional oncology and palliative care centre in Timișoara, Romania, between July and December 2024. Data were collected using standardized self-report questionnaires administered during routine clinical contact, complemented by extraction of relevant clinical information from medical records.

### 2.2. Participants

**Figure 1:**
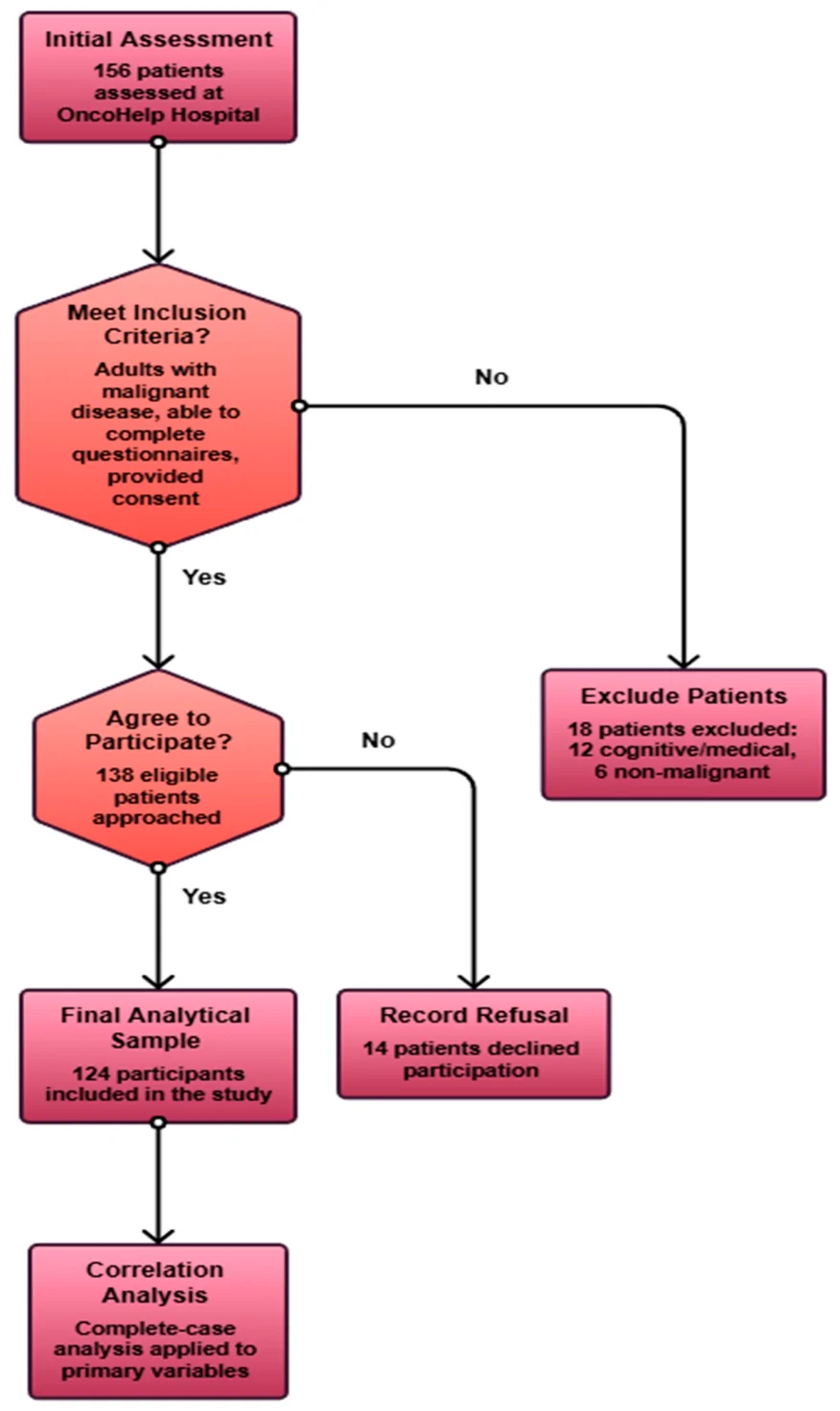
Patients flow-chart recruitment.

During the data collection period (July–December 2024), a total of 156 patients attending the oncology outpatient clinic and inpatient ward at OncoHelp Hospital were assessed for eligibility. Of these, 18 were excluded because they did not meet the inclusion criteria (inability to complete questionnaires due to cognitive impairment or acute medical deterioration, *n* = 12; non-malignant primary diagnosis, *n* = 6), leaving 138 eligible patients who were approached for participation. Of these, 14 declined participation, yielding a final analytical sample of 124 participants. Eligible participants were adults diagnosed with malignant disease who were able to understand and complete the questionnaires and who provided informed consent (Figure 1).

### 2.3. Measures

Sociodemographic variables included age, sex, residence (urban/rural), educational attainment, and marital status. Anthropometric status was assessed using body mass index (BMI, kg/m^2^) and categorized as underweight, normal weight, overweight, or obese. Clinical variables included cancer type, years since diagnosis, disease stage (localized, metastatic, terminal, remission), comorbidity burden, Eastern Cooperative Oncology Group (ECOG) performance status, and treatment modalities received (surgery, chemotherapy, radiotherapy, immunotherapy, targeted therapy, biologic therapy, and supportive care).

### 2.4. Spiritual Well-Being

Spiritual well-being was assessed using the 12-item Functional Assessment of Chronic Illness Therapy–Spiritual Well-Being scale (FACIT-Sp-12). Items were rated on a 4-point Likert scale, with higher scores indicating greater spiritual well-being. Total scores were computed according to the instrument scoring approach, with negatively worded items reverse-coded as appropriate [5]. We included FACIT-Sp-12 as one measure of spiritual well-being because of its widespread use in studies examining the medical impact of low spiritual well-being (Table S1) [18,19,20].

The original English version of the FACIT-Sp-12 was translated into Romanian using standard forward-backward methodology. First, two bilingual health professionals independently translated the instrument into Romanian. These versions were synthesized into a single draft, which was then back-translated into English by a third independent translator to ensure conceptual equivalence. Minor discrepancies were resolved by consensus. In the present study, the Romanian version demonstrated acceptable internal consistency (Cronbach’s α = 0.732), comparable to the original English validation.

### 2.5. Religiosity

Religious commitment was measured using the Religious Commitment Inventory (RCI-10), a 10-item instrument assessing the extent to which religious beliefs and practices are central to an individual’s life. Items were rated on a 5-point Likert scale, with higher scores indicating greater religious commitment. In addition, religious and spiritual characteristics were collected, including religious affiliation, worship frequency, importance of faith, perceived sources of meaning, perceived spiritual support during illness, influence of illness on spiritual practices, and perceived change in spiritual beliefs (Table S2) [21].

As a standardized Romanian version was not available, the original English instrument was translated using a standard forward-backward translation procedure to ensure linguistic and cultural equivalence. The scale demonstrated excellent internal consistency in the present study sample (Cronbach’s α = 0.957).

### 2.6. Psychological Distress

Anxiety and depression symptoms were assessed using the Hospital Anxiety and Depression Scale (HADS), a 14-item instrument comprising two 7-item subscales (Anxiety and Depression), each scored from 0 to 21. Higher scores indicate greater symptom burden. Scores were categorized using standard cut-offs: 0–7 (normal), 8–10 (borderline), and 11–21 (clinically significant) (Table S3) [22]. We used a Romanian validated questionnaire form [23].

### 2.7. Quality of Life

Health-related quality of life was evaluated using the European Organisation for Research and Treatment of Cancer Quality of Life Questionnaire (EORTC QLQ-C30, version 3.0). This 30-item instrument comprises five functional scales (physical, role, emotional, cognitive, and social), three symptom scales (fatigue, nausea/vomiting, and pain), six single items assessing common cancer symptoms, and a Global Health Status/QoL scale. Items 1–28 are rated on a 4-point Likert scale (1 = “Not at all” to 4 = “Very much”), while the two global items (Q29 and Q30) utilize a 7-point scale (1 = “Very poor” to 7 = “Excellent”). All scores were linearly transformed to a 0–100 scale according to the EORTC scoring manual, where higher scores on functional and global scales represent better functioning/QoL, and higher scores on symptom scales indicate greater symptom burden (Tables S4 and S5). We utilized the official Romanian translation provided by the EORTC Quality of Life Group, which has demonstrated linguistic and psychometric equivalence to the original English version in international trials [24].

### 2.8. Statistical Analysis

Data management and statistical analyses were conducted using IBM SPSS Statistics, version 26.0 (IBM Corp., Armonk, NY, USA). Descriptive statistics were used to summarize patient demographics and clinical characteristics, with continuous variables presented as means (SD) or medians (IQR) and categorical variables as frequencies and percentages. The normality of data distribution was assessed using the Shapiro–Wilk test; given the non-Gaussian distribution of primary variables (*p* < 0.001), non-parametric tests were employed. A formal a priori power calculation was not performed for this exploratory, cross-sectional study. The sample of 124 participants represents all consecutive eligible patients enrolled during the data collection period and was determined by feasibility. Post hoc sensitivity analysis indicates that, at α = 0.05 and 80% power, our sample is adequate to detect medium-sized correlations (r ≥ 0.25) but may be insufficient to detect small effects (r < 0.15).

Associations between spiritual well-being (FACIT-Sp-12), religious commitment (RCI-10), psychological distress (HADS), and quality of life (EORTC QLQ-C30) were initially evaluated using Spearman’s rank correlation coefficients (r). To examine the predictors of patient outcomes, a two-step regression approach was utilized. First, univariate linear regression was performed for all demographic and clinical variables. Variables demonstrating significant or near-significant associations (*p* < 0.10) in the univariate analysis (FACIT-Sp-12, RCI-10, and ECOG status) were then entered into multivariable linear regression models to identify independent predictors. All statistical tests were two-sided, with significance set at *p* value < 0.05.

### 2.9. Ethical Considerations

The study was conducted in accordance with the Declaration of Helsinki and was approved by the Ethics Committee of OncoHelp (1657/16.07.2024). All participants provided informed consent prior to questionnaire completion.

### 2.10. Reliability Analysis

Internal consistency of the study instruments was evaluated using Cronbach’s alpha coefficients (α) based on complete-case data. The Hospital Anxiety and Depression Scale (HADS) demonstrated acceptable internal consistency overall, with an α of 0.760 for the total scale. Subscale reliability was acceptable for the depression subscale (α = 0.757) and moderate for the anxiety subscale (α = 0.684).

The FACIT-Sp-12 showed acceptable internal consistency for the total scale (α = 0.732). Subscale-level analysis revealed varying degrees of internal consistency across its three domains: Faith demonstrated high reliability (α = 0.859), while the Meaning (α = 0.532) and Peace (α = 0.541) subscales showed lower coefficients, likely reflecting the diverse conceptualizations of these existential constructs in the Romanian cultural context.

The Religious Commitment Inventory (RCI-10) demonstrated excellent total internal consistency (α = 0.957). Subscale reliability was high for both the Intrapersonal (private faith; α = 0.941) and Interpersonal (public/social faith; α = 0.758) dimensions.

## 3. Results

The cohort included 124 patients with a mean age of 65 years (IQR 53–71), a balanced gender distribution (49% women) and almost equal urban-rural residence (Table 1). The median BMI was 25.4 kg (IQR 22.2–27.6), with 81 percent of patients classified as either normal weight or overweight. Education was divided between secondary (19%), vocational (21), upper secondary (38) and upper university (23%). The majority of the participants were married (67 percent). Religious affiliation was predominantly Orthodox (82%), followed by Catholic (8.1%) and Pentecostal (4.0%). Worship attendance was distributed across weekly (26%), regularly (26%), monthly (12%), occasionally (26%), and rarely (10%). Faith was highly valued, with 73 (59%) reporting it as “very important” and 96 (77%) identifying faith as their primary source of meaning. Spiritual support during illness was sought by 87 (70%) patients. Illness influenced spiritual practices in most participants: 66 (53%) reported intensified practices, 44 (35%) no change, 8 (6.5%) new practices, and 6 (4.8%) decreased practices.

Multimorbidity was common, with 45 (36%) patients having 1 comorbidity, 56 (45%) having 2–3 comorbidities, and 23 (19%) having ≥4 comorbidities (Table 1). Median time since diagnosis was 2.0 years (IQR 1.0–5.0). The most frequent cancer types were colorectal (31%), breast (19%), and broncho-pulmonary (12%). Disease stage was predominantly advanced: 70 (56%) had metastatic disease, 34 (27%) localized disease, 6 (4.8%) terminal illness, 5 (4.0%) metastatic with terminal features, and 9 (7.3%) were in remission. ECOG performance status showed 50 (40%) at ECOG 0, 39 (31%) at ECOG 1, 18 (15%) at ECOG 2, 9 (7.3%) at ECOG 3, and 8 (6.5%) at ECOG 4. Treatment modalities included chemotherapy in 98 (79%), surgery in 39 (31%), radiotherapy in 28 (23%), immunotherapy in 15 (12%), targeted therapy in 11 (8.9%), supportive care in 13 (10%), and biologic therapy in 3 (2.4%) (Table 1).

The median HADS Anxiety and Depression Total scores were 6.0 (IQR 4.0–8.0) and 7.0 (IQR 4.0–11.0), respectively. Based on standard categorical cut-offs, 69% of patients had normal anxiety symptoms, 18% borderline anxiety symptoms, and 14% clinically significant anxiety symptoms; for depression, the corresponding proportions were 56% normal, 16% borderline, and 28% clinically significant depressive symptoms. Clinically significant depressive symptoms were therefore approximately twice as prevalent as clinically significant anxiety symptoms in this cohort.

**Figure 2:**
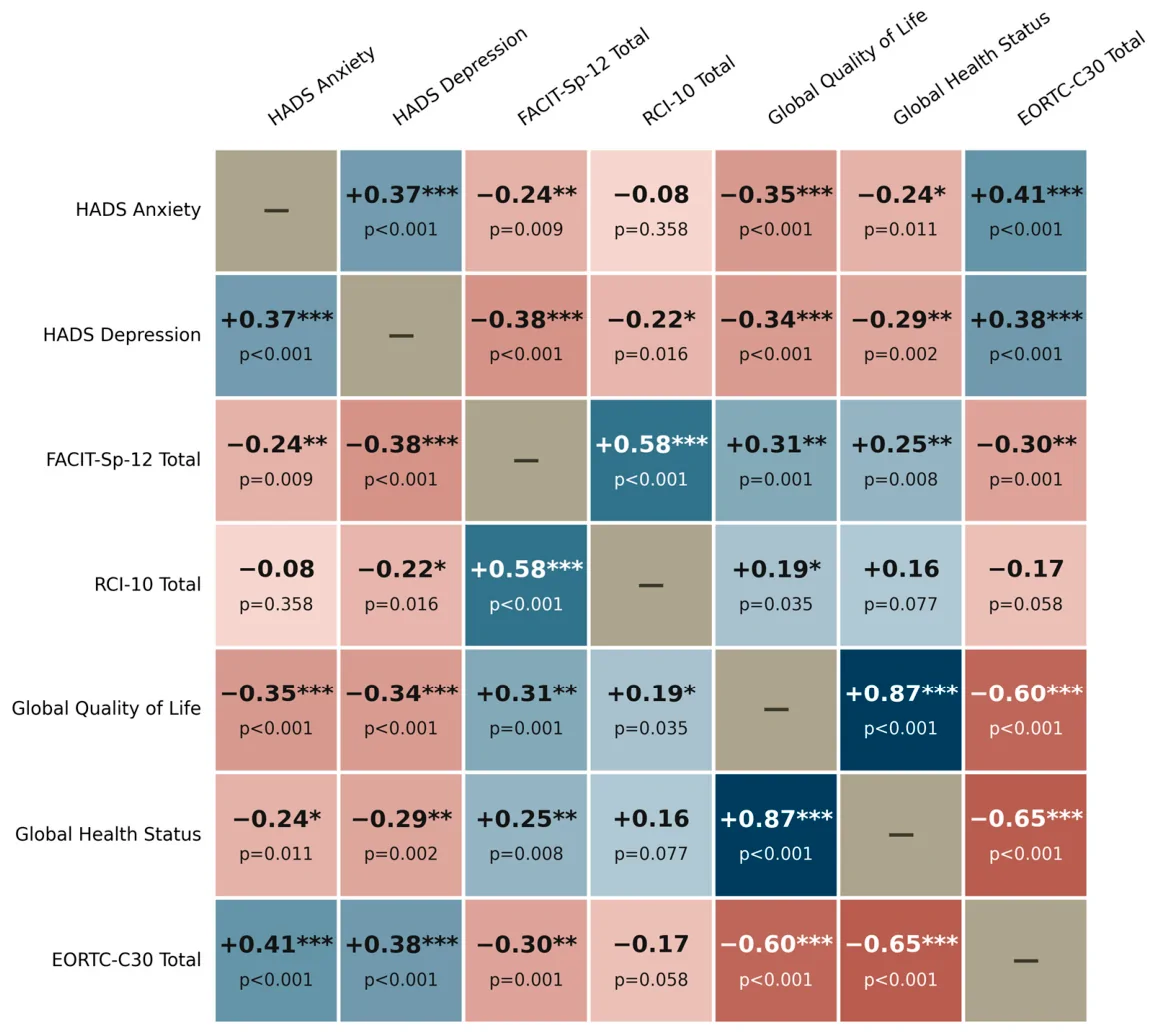
Spearman correlation matrix among psychological distress, spiritual well-being, religious commitment, and quality-of-life measures. (Heatmap of pairwise Spearman correlation coefficients (r) among the seven primary study variables. Cell color reflects the direction and strength of each correlation (blue = positive, coral = negative; darker shading = stronger association). Values shown are r and FDR-adjusted *p*-values (Benjamini–Hochberg correction for 21 comparisons); asterisks denote significance levels (* *p* < 0.05, ** *p* < 0.01, *** *p* < 0.001). *n*= 124.).

Figure 2 presents the Spearman correlation matrix among the seven primary study variables. Spiritual well-being (FACIT-Sp-12) was inversely correlated with both anxiety (r = −0.24, *p* = 0.009) and depression (r = −0.38, *p* < 0.001), and positively correlated with global quality of life (r = 0.31, *p* = 0.001) and global health status (r = 0.25, *p* = 0.008); religious commitment (RCI-10), despite a strong correlation with FACIT-Sp-12 (r = 0.58, *p* < 0.001), showed a weaker and non-significant association with anxiety (r = −0.08, *p* = 0.358) and only a modest association with depression (r = −0.22, *p* = 0.016). As expected, the two global quality-of-life indices were very strongly correlated with one another (r = 0.87, *p* < 0.001) and both were strongly inversely correlated with overall symptom burden (EORTC-C30 Total, r = −0.60 and −0.65, respectively), while HADS Anxiety and Depression were themselves moderately correlated (r = 0.37, *p* < 0.001).

In multivariable linear regression analysis, spiritual well-being (FACIT-Sp-12) showed a moderate inverse association with anxiety (β = −0.25; *p* = 0.030, q = 0.037) in a model accounting for 11.5% of variance (R^2^ = 0.115; adjusted R^2^ = 0.070). FACIT-Sp-12 was similarly associated with depression (β = −0.29; *p* = 0.013, q = 0.046), with the overall model explaining 10.8% of variance (R^2^ = 0.108; adjusted R^2^ = 0.063). Neither association survived strict correction for multiple comparisons in this fully adjusted model, though FACIT-Sp-12 remained the strongest correlate of both outcomes among the covariates tested. Global quality of life was more fully accounted for by the model predictors (R^2^ = 0.264; adjusted R^2^ = 0.213), explaining 26.4% of variance, and was independently associated with impaired performance status (ECOG, β = −0.27; *p* = 0.006, q = 0.048) and depressive symptoms (β = −0.22; *p* = 0.014, q = 0.039) both narrowly outside the conventional q < 0.05 threshold after correction. Anxiety, disease stage, and demographic variables were not independently associated with global quality of life after accounting for physical status and depressive symptoms (Table 2).

## 4. Discussion

The present study examined the associations between spiritual well-being, religiosity, psychological distress, and quality of life (QoL) in a cohort of Romanian cancer patients. The results yield three primary insights: (1) spiritual well-being (SWB) was significantly and independently associated with lower levels of anxiety and depression; (2) while religious commitment (religiosity) was high and often intensified by the illness experience, it was not independently associated with mental health outcomes in multivariable models; and (3) global quality of life was independently associated with both physical performance status (ECOG) and depressive symptom burden, rather than with spiritual or religious factors.

A critical finding of this study is the distinction between spiritual well-being (measured by FACIT-Sp-12) and religious commitment (measured by RCI-10). While bivariate analysis showed that both constructs were correlated with lower depression, only SWB remained significantly and independently associated with depression in the adjusted regression models. This suggests that religious practice and spiritual well-being are distinct constructs with different associations with mental health.

Grossoehme et al. (2020) found, in adolescents and young adults with cancer, that religious behaviors were inversely associated with anxiety and depression (β = −7.94 for anxiety; β = −10.49 for depression) only when mediated by a sense of meaning and peace [12], and Schultz et al. (2017) reported that among the FACIT-Sp subscales, only “Peace” reliably distinguished spiritual distress (AUC = 0.73), whereas “Faith” showed no significant predictive ability [13]. A recent meta-analysis by Martinez-Calderon et al. (2025) found that while SWB was significantly correlated with reduced cancer-related fatigue (r = −0.37), the correlation between faith specifically and fatigue was not statistically significant (*p* = 0.36) [11].

Consistent with previous literature, SWB showed a stronger inverse association with depression (r = −0.380) than with anxiety (r = −0.245). In our sample, clinically significant depression (28%) was notably higher than anxiety (14%). This inverse relationship mirrors findings by Torres-Blasco et al. (2024) in Latino populations, where SWB was a significant negative predictor of hopelessness (β = −0.152) and depression (β = −0.161) [14]. Torres-Blasco et al. suggest that for culturally religious groups (like Latinos and, arguably, Romanians), SWB is associated with lower existential hopelessness, which is itself linked to depressive symptoms.

Conversely, the relationship between SWB and anxiety appears more complex. Londoudi et al. (2024) found that Fear of Cancer Recurrence (FCR) is a distinct pathway to anxiety, noting a negative correlation between SWB and FCR (r = −0.36) [25]. It is possible that while SWB mitigates existential depression, the immediate, somatic arousal of anxiety (measured by HADS-A) is less responsive to spiritual reframing and more dependent on disease trajectory and physical symptoms.

Regarding Quality of Life, our multivariable analysis identified ECOG performance status as the variable most strongly and independently associated with Global Health Status/QoL, whereas the univariate association observed for SWB (r = 0.306) did not persist after adjustment. This finding contrasts with Biji et al. (2025), who reported a strong positive correlation (r = 0.766) between SWB and Global QoL in Indian palliative care patients [8]. The discrepancy may be explained by the high burden of advanced disease in our cohort (56% metastatic).

Our results are more aligned with Mendes et al. (2023), who found that symptom overload and functional decline were the primary drivers of reduced performance (r = −0.317) in palliative settings [26]. This suggests a hierarchy of needs in advanced cancer: while spirituality is vital for coping, perceived Global Quality of Life is heavily anchored in physical functionality. When physical limitations are severe (high ECOG scores), the buffering effect of SWB on overall QoL scores may be diminished.

### 4.1. Clinical Implications

These findings have direct implications for oncological care in Romania. Routine screening should look beyond religious affiliation. As Schultz et al. noted, general distress thermometers often fail to identify spiritual distress (Sensitivity 41%), necessitating specific tools that assess “Peace” and “Meaning” [13].

It may be useful to examine whether interventions that help patients move from ritual religious observance toward an internalized sense of meaning and peace are associated with improved psychological outcomes; given the cross-sectional design of the present study, such approaches would need to be tested formally, for example through longitudinal or randomized intervention designs, before being incorporated into clinical guidelines. Momennasab et al. (2024) demonstrated in a randomized clinical trial that spiritual group therapy significantly improved general health (*p* = 0.016) and emotional functioning (*p* = 0.029) in breast cancer patients [27]. Given the high baseline religiosity in our population, such interventions could be highly effective if they move beyond ritual to address existential processing, helping patients find meaning within their suffering.

It is possible that religious commitment confers psychological benefit through pathways not captured directly by our distress measures for instance, by reducing loneliness through social and communal participation. Public health messaging has similarly positioned religious engagement as one of several means of addressing loneliness [28]. As our study did not directly assess loneliness or social support, future research in this population should examine whether these constructs mediate the relationship between religious commitment and psychological well-being.

### 4.2. Limitations

A number of limitations should be noted. First, the cross-sectional design prevents causal inference; it is not possible to determine whether higher SWB reduces depression or whether less depressed patients may maintain higher SWB. Second, the use of a convenience sample from a single tertiary cancer centre introduces a risk of selection bias; patients who agree to take part may systematically differ from those who decline, for example by being more mentally engaged, more emotionally resistant or less symptomatic. Third, the single-centre setting in Eastern Europe, with its small sample size, limits the generalisation to non-orthodox populations. Fourth, several variables related to spirituality and religiosity showed a pronounced ceiling effect, reflecting the high baseline religiosity of this cohort: 123 of 124 participants (99%) reported a positive change in spiritual beliefs due to illness, importance of faith was rated as ‘important’ or ‘very important’ by 97.6% of participants, and FACIT-Sp-12 scores averaged 75% of the maximum possible score. This restricted range may have attenuated the observed associations between spirituality-related variables and distress outcomes, and the true strength of these relationships may be underestimated relative to more religiously heterogeneous populations. Fifth, the Meaning (α = 0.532) and Peace (α = 0.541) subscales of the FACIT-Sp-12 showed suboptimal internal consistency, in contrast to the Faith subscale (α = 0.859); as only the total score was used in regression modelling, we cannot attribute the observed associations specifically to meaning and peace as distinct from faith, and subscale-level analyses in larger samples are needed to clarify this. Sixth, neither the FACIT-Sp-12 nor the RCI-10 has been formally validated psychometrically (e.g., via confirmatory factor analysis) in a Romanian oncology population; forward-backward translation and internal consistency analysis were performed, but structural and cross-cultural validity were not formally established. Finally, the use of full-case analysis may introduce bias when the absence is related to the patient’s medical distress or functional status.

## 5. Conclusions

In this cohort of Romanian cancer patients, spiritual well-being—especially sense of purpose and peace—was a strong independent predictor of reduced depression and anxiety, even after adjustment for religious adherence and functional status. Religious adherence alone has not been shown to be independent of mental health outcomes, underlining the clinical need to distinguish religious behaviour from spiritual well-being in the assessment of psychological risk. Physical functional status and depressive symptom burden, rather than religious commitment or spiritual well-being, remained most strongly associated with quality of life in this population with advanced disease. Due to the cross-sectional design, these findings are best interpreted as hypothesis-based; future studies are needed to determine whether psychological interventions can make a meaningful difference in the psychological outcomes of cancer treatment.

## Figures and Tables

**Table 1:** Baseline characteristics.

Variable	Values	Variable	Values
**Age, years**	65 (53–71)	BMI, kg/m^2^	25.4 (22.2–27.6)
**BMI category**	Underweight 8 (6.5%) Normal 51 (41%) Overweight 49 (40%) Obese 16 (13%)	Marital status	Single 11 (8.9%) Married 83 (67%) Divorced 13 (10%) Widowed 17 (14%)
**Education**	Middle school 23 (19%) Vocational 26 (21%) High school 47 (38%) Higher education 28 (23%)	Residence	Urban 66 (53%) Rural 58 (47%)
**Gender**	Female 61 (49%) Male 63 (51%)	Religious affiliation	Orthodox 102 (82%) Catholic 10 (8.1%) Pentecostal 5 (4.0%) Others ≤1% each
**Worship attendance**	Daily 1 (0.8%) Weekly 32 (26%) Regularly 32 (26%) Monthly 15 (12%) Occasionally 32 (26%) Rarely 12 (9.7%)	Importance of faith	Very important 73 (59%) Important 48 (39%) Not important 3 (2.4%)
**Meaning in life (short)**	Faith 96 (77%) Family 24 (19%) Other 4 (3.2%)	Spiritual support during illness	Yes 87 (70%)
**Influence on spiritual practices**	Intensified 66 (53%) No change 44 (35%) New practices 8 (6.5%) Decreased 6 (4.8%)	Change in spiritual beliefs	Positive 123 (99%) Negative 1 (0.8%)
**Comorbidities**	1: 45 (36%) 2–3: 56 (45%) ≥4: 23 (19%)	Years since diagnosis	2.0 (1.0–5.0)
**Cancer type (common)**	Colorectal 38 (31%) Breast 23 (19%) Pulmonary 15 (12%)	Stage of disease	Localized 34 (27%) Metastatic 70 (56%) Terminal 6 (4.8%) Remission 9 (7.3%)
**ECOG status**	0: 50 (40%) 1: 39 (31%) 2: 18 (15%) 3: 9 (7.3%) 4: 8 (6.5%)	Treatments received	Surgery 39 (31%) Chemotherapy 98 (79%) Radiotherapy 28 (23%) Immunotherapy 15 (12%) Targeted 11 (8.9%) Supportive 13 (10%) Biologic 3 (2.4%)

Abbreviations: BMI, body mass index (kg/m^2^); ECOG, Eastern Cooperative Oncology Group performance status (0 = fully active; 1 = restricted in strenuous activity; 2 = ambulatory, capable of self-care; 3 = limited self-care; 4 = completely disabled); IQR, interquartile range.

**Table 2:** Multivariable linear regression models for HADS Anxiety, HADS Depression, and Global Quality of Life, adjusted for spiritual well-being, religious commitment, functional status, and demographic/clinical covariates.

Predictor	HADS Anxiety				HADS Depression				Global QoL (with HADS)			
	β (95% CI)	*p*	q	VIF	β (95% CI)	*p*	q	VIF	β (95% CI)	*p*	q	VIF
Age	−0.18 (−0.36, 0.00)	0.048	0.16	1.1	−0.05 (−0.23, 0.14)	0.6	0.99	1.1	0.04 (−0.13, 0.21)	0.6	0.64	1.1
RCI-10 Total	0.09 (−0.13, 0.31)	0.4	0.50	1.7	0.00 (−0.23, 0.22)	>0.9	0.99	1.7	0.08 (−0.13, 0.28)	0.4	0.57	1.7
ECOG Performance Status	0.19 (−0.01, 0.40)	0.067	0.16	1.4	0.08 (−0.12, 0.29)	0.4	0.99	1.4	−0.27 (−0.47, −0.08)	0.006	0.048	1.5
FACIT-Sp-12 Total	−0.25 (−0.48, −0.02)	0.030	0.037	1.7	−0.29 (−0.52, −0.06)	0.013	0.046	1.7	0.11 (−0.11, 0.32)	0.3	0.50	1.9
Sex: Female (ref. Male)	0.15 (−0.21, 0.52)	0.4	0.50	1.1	−0.02 (−0.38, 0.35)	>0.9	0.99	1.1	−0.20 (−0.53, 0.14)	0.2	0.50	1.1
Stage: Metastatic/Terminal (ref. Localized/Remission)	−0.24 (−0.64, 0.17)	0.3	0.44	1.3	0.11 (−0.30, 0.52)	0.6	0.99	1.3	−0.10 (−0.48, 0.27)	0.6	0.64	1.3
HADS Anxiety Total	—	—	—	—	—	—	—	—	−0.12 (−0.30, 0.05)	0.2	0.50	1.2
HADS Depression Total	—	—	—	—	—	—	—	—	−0.22 (−0.39, −0.04)	0.014	0.039	1.2
Model fit	R^2^ = 0.115; Adj. R^2^ = 0.070				R^2^ = 0.108; Adj. R^2^ = 0.063				R^2^ = 0.264; Adj. R^2^ = 0.213			

## Data Availability

The data presented in this study are available upon reasonable request from the corresponding author. The data are not publicly available due to privacy and ethical restrictions.

## Supplementary Materials

The following supporting information can be downloaded at: https://www.mdpi.com/article/10.3390/cancers18172840/s1. Table S1: FACIT – SP12 distribution; Table S2: RCI distribution; Table S3: Hospital Anxiety and Depression Scale (HADS) – Item-Level Distributions; Table S4: Distribution of EORTC QLQ-C30 Item Responses; Table S5: Distribution of the rest of EORTC QLQ-C30 Item Responses.
