# Supplementary material for: Spiritual Well-Being, Religiosity, Psychological Distress and Quality of Life in Adults with Cancer: A Cross-Sectional Study in a Romanian Oncology and Palliative Care Centre
Source: Cancers (Basel). 2026 Sep 2;18(17):2840. doi: 10.3390/cancers18172840 (PMC13565442; doi:10.3390/cancers18172840)
Supplement: Supplementary file 1 [file cancers-18-02840-s001.zip › cancers-4507501-supplementary.pdf]

Table S1. FACIT – SP12 distribution

Item	Paraphrased description (English)	1 n (%)	2 n (%)	3 n (%)	4 n (%)
**SP12 Q1**	I feel a sense of inner peace.	8 (6.5%)	26 (21%)	39 (31%)	51 (41%)
**SP12 Q2**	I feel that I have a reason to go on living.	4 (3.2%)	5 (4.0%)	25 (20%)	90 (73%)
**SP12 Q3**	I feel that my life has been useful and productive.	8 (6.5%)	14 (11%)	28 (23%)	74 (60%)
**SP12 Q4**	I have difficulty feeling calm and at peace.	77 (62%)	19 (15%)	17 (14%)	11 (8.9%)
**SP12 Q5**	I experience a clear sense of purpose in my life.	7 (5.6%)	10 (8.1%)	24 (19%)	83 (67%)
**SP12 Q6**	I can draw on inner spiritual resources for comfort when I need them.	11 (8.9%)	16 (13%)	31 (25%)	66 (53%)
**SP12 Q7**	My illness has deepened or strengthened my spiritual or religious beliefs.	17 (14%)	26 (21%)	29 (23%)	52 (42%)
**SP12 Q8**	I feel distant or disconnected from God or from what I consider sacred.	90 (73%)	15 (12%)	7 (5.6%)	12 (9.7%)
**SP12 Q9**	I am confident that, whatever happens with my illness, things will ultimately be all right.	11 (8.9%)	16 (13%)	27 (22%)	70 (56%)
**SP12_10**	I find emotional comfort in my faith or spiritual beliefs.	10 (8.1%)	17 (14%)	27 (22%)	70 (56%)
**SP12_11**	I draw strength from my faith or spiritual beliefs.	11 (8.9%)	19 (15%)	25 (20%)	69 (56%)
**SP12_12**	I feel that my life has meaning and purpose.	8 (6.5%)	15 (12%)	29 (23%)	72 (58%)

Table S2. RCI distribution

Item	Variable	1 n (%)	2 n (%)	3 n (%)	4 n (%)	5 n (%)
**RCI10 Q1**	I invest time reading or learning about my faith.	35 (28%)	19 (15%)	16 (13%)	17 (14%)	37 (30%)
**RCI10 Q2**	I contribute time or resources to my religious community.	34 (27%)	20 (16%)	19 (15%)	17 (14%)	34 (27%)
**RCI10 Q3**	I actively seek to grow in my understanding of my religion.	22 (18%)	21 (17%)	26 (21%)	18 (15%)	37 (30%)
**RCI10 Q4**	My religion helps me find meaning and answers to fundamental questions about life.	15 (12%)	10 (8.1%)	17 (14%)	19 (15%)	63 (51%)
**RCI10 Q5**	My overall approach to life is guided by my religious beliefs.	22 (18%)	8 (6.5%)	15 (12%)	20 (16%)	59 (48%)
**RCI10 Q6**	I value spending time with others who share my faith at religious activities.	28 (23%)	16 (13%)	19 (15%)	15 (12%)	46 (37%)
**RCI10 Q7**	I am willing to make sacrifices in order to live according to my religion.	30 (24%)	17 (14%)	15 (12%)	20 (16%)	42 (34%)
**RCI10 Q8**	I try to apply my religious beliefs consistently in my everyday dealings and relationships.	21 (17%)	19 (15%)	18 (15%)	27 (22%)	39 (31%)
**RCI10 Q9**	I seek God's guidance when making important decisions.	43 (35%)	20 (16%)	22 (18%)	12 (9.7%)	27 (22%)
**RCI10 Q10**	I remain informed and involved in the activities of my religious community.	21 (17%)	19 (15%)	18 (15%)	27 (22%)	39 (31%)

Table S3. Hospital Anxiety and Depression Scale (HADS) – Item-Level Distributions

Item	Variable	0	1	2	3
**HADS-Dep 1**	Reduced interest or enjoyment	73 (59%)	19 (15%)	17 (14%)	15 (12%)
**HADS-Dep 2**	Feeling slowed down or lacking energy	60 (48%)	27 (22%)	27 (22%)	10 (8.1%)
**HADS-Dep 3**	Loss of enjoyment in things previously pleasurable	41 (33%)	39 (31%)	29 (23%)	15 (12%)
**HADS-Dep 4**	Difficulty seeing positive aspects of life	38 (31%)	35 (28%)	32 (26%)	19 (15%)
**HADS-Dep 5**	Reduced interest in appearance / daily activities	27 (22%)	24 (19%)	40 (32%)	33 (27%)
**HADS-Dep 6**	Expecting less enjoyment from the future	56 (45%)	24 (19%)	25 (20%)	19 (15%)
**HADS-Dep 7**	Hopelessness / feeling life is not worthwhile	70 (56%)	21 (17%)	14 (11%)	19 (15%)
**HADS-Anx 1**	Feeling tense or wound up	60 (48%)	39 (31%)	13 (10%)	11 (8.9%)
**HADS-Anx 2**	Worrying thoughts / difficulty relaxing	72 (58%)	26 (21%)	14 (11%)	11 (8.9%)
**HADS-Anx 3**	Sudden feelings of fear	54 (44%)	42 (34%)	13 (10%)	14 (11%)
**HADS-Anx 4**	Feeling panicky or overwhelmed	10 (8.1%)	27 (22%)	27 (22%)	60 (48%)
**HADS-Anx 5**	Restlessness / inability to sit still	77 (62%)	25 (20%)	13 (10%)	9 (7.3%)
**HADS-Anx 6**	Physical symptoms of anxiety (e.g., stomach sensation)	47 (38%)	36 (29%)	26 (21%)	15 (12%)
**HADS-Anx 7**	Feeling frightened for no clear reason	88 (71%)	21 (17%)	8 (6.5%)	7 (5.6%)

Table S4. Distribution of EORTC QLQ-C30 Item Responses

Item	Paraphrased Description	1 n (%)	2 n (%)	3 n (%)	4 n (%)
**Q1**	Trouble doing strenuous activities	19 (15.3%)	19 (15.3%)	35 (28.2%)	51 (41.2%)
**Q2**	Trouble taking a long walk	53 (42.7%)	35 (28.2%)	16 (12.9%)	20 (16.1%)
**Q3**	Trouble taking a short walk	28 (22.6%)	42 (33.9%)	24 (19.4%)	30 (24.2%)
**Q4**	Need to stay in bed or chair during the day	78 (62.9%)	19 (15.3%)	12 (9.7%)	15 (12.1%)
**Q5**	Need help with eating, dressing, or washing	29 (23.4%)	35 (28.2%)	26 (21.0%)	34 (27.4%)
**Q6**	Limited in doing either your work or other daily activities	44 (35.5%)	28 (22.6%)	29 (23.4%)	23 (18.5%)
**Q7**	Limited in pursuing your hobbies or other leisure activities	25 (20.2%)	17 (13.7%)	32 (25.8%)	50 (40.3%)
**Q8**	Were you short of breath?	93 (75.0%)	16 (12.9%)	8 (6.5%)	7 (5.6%)
**Q9**	Have you had pain?	51 (41.1%)	28 (22.6%)	27 (21.8%)	18 (14.5%)
**Q10**	Did you need to rest?	46 (37.1%)	37 (29.8%)	22 (17.7%)	19 (15.3%)
**Q11**	Have you had trouble sleeping?	39 (31.5%)	37 (29.8%)	27 (21.8%)	21 (16.9%)
**Q12**	Have you felt weak?	43 (34.7%)	37 (29.8%)	22 (17.7%)	22 (17.7%)
**Q13**	Have you lacked appetite?	56 (45.2%)	26 (21.0%)	25 (20.2%)	17 (13.7%)
**Q14**	Have you felt nauseated?	68 (55.3%)	26 (21.1%)	17 (13.8%)	12 (9.8%)
**Q15**	Have you vomited?	89 (71.8%)	19 (15.3%)	8 (6.5%)	8 (6.5%)
**Q16**	Have you been constipated?	71 (57.3%)	21 (16.9%)	19 (15.3%)	13 (10.5%)
**Q17**	Have you had diarrhea?	89 (71.8%)	19 (15.3%)	10 (8.1%)	6 (4.8%)
**Q18**	Were you tired?	32 (26.0%)	35 (28.5%)	32 (26.0%)	24 (19.5%)
**Q19**	Did pain interfere with your daily activities?	64 (51.6%)	22 (17.7%)	25 (20.2%)	13 (10.5%)
**Q20**	Have you had difficulty in concentrating on things?	80 (64.5%)	27 (21.8%)	11 (8.9%)	6 (4.8%)
**Q21**	Did you feel tense?	57 (46.0%)	34 (27.4%)	20 (16.1%)	13 (10.5%)
**Q22**	Did you worry?	55 (44.4%)	39 (31.5%)	20 (16.1%)	10 (8.1%)
**Q23**	Did you feel irritable?	73 (58.9%)	24 (19.4%)	20 (16.1%)	7 (5.6%)
**Q24**	Did you feel depressed?	78 (62.9%)	24 (19.4%)	16 (12.9%)	6 (4.8%)
**Q25**	Have you had difficulty remembering things?	70 (56.5%)	35 (28.2%)	13 (10.5%)	6 (4.8%)

**Q26**	Has your physical condition/treatment interfered with family life?	66 (53.2%)	27 (21.8%)	23 (18.5%)	8 (6.5%)
**Q27**	Has your physical condition/treatment interfered with social activities?	73 (58.9%)	21 (16.9%)	21 (16.9%)	9 (7.3%)
**Q28**	Has your physical condition/treatment caused financial difficulties?	85 (68.5%)	19 (15.3%)	14 (11.3%)	6 (4.8%)

Table S5. Distribution of the rest of EORTC QLQ-C30 Item Responses

Item #	Description	1–2 (Poor)	3	4	5	6	7
**Q29**	Rate your overall health during the past week	9 (7.2%)	12 (9.7%)	16 (12.9%)	34 (27.4%)	32 (25.8%)	21 (16.9%)
**Q30**	Rate your overall quality of life during the past week	9 (7.2%)	14 (11.3%)	10 (8.1%)	32 (25.8%)	37 (29.8%)	22 (17.7%)
